# *Polyporus ulleungus* mycelia cultured in MEB medium produce metabolites with anticancer property

**DOI:** 10.7150/jca.89059

**Published:** 2024-01-01

**Authors:** Yun Haeng Lee, Myeong Uk Kuk, Moon Kyoung So, Hyon Jin Park, Eun Seon Song, Jiho Park, Jee hee Yoon, Hyung Wook Kwon, Jaehyuk Choi, Joon Tae Park

**Affiliations:** 1 Division of Life Sciences, College of Life Sciences and Bioengineering, Incheon National University, Incheon, Korea.; 2 Convergence Research Center for Insect Vectors, Incheon National University, Incheon 22012, Korea.

**Keywords:** *Polyporus ulleungus*, mycelium culture extract, drug screening

## Abstract

Cancer cells are characterized by apoptosis evasion and uncontrolled cell cycle progression. To combat these characteristics, efforts have been made to find novel natural-source anticancer compounds. The aim of this work is to find new anticancer compounds in *Polyporus ulleungus* (*P. ulleungus*) mycelial culture extracts. *P. ulleungus* mycelium was cultured on four individual media (DYB, MEB, MYB, and PDB) and four extracts were generated from the mycelium culture media. Extracts of *P. ulleungus* mycelium cultured in MEB medium (pu-MEB) significantly reduced cancer cell growth by triggering apoptosis and S phase arrest. Furthermore, the anticancer effects of pu-MEB were not confined to one type of cancer. Taken together, our results confirmed that *P. ulleungus* mycelia cultured in MEB medium produce metabolites that exhibit anticancer properties. Development of an optimal medium for *P. ulleungus* mycelium through optimization of medium components will enable *P. ulleungus* mycelium to produce metabolites with more anticancer efficacy.

## Introduction

Mushrooms are not only a source of nutrients but are also recognized as health-promoting foods as they produce physiologically active substances 1. Mushrooms synthesize primary metabolites that are essential for mushroom growth and secondary metabolites that are produced in response to various environmental stimuli 2. Secondary metabolites exhibit immunomodulatory, anticancer, antibacterial, anti-HIV, and antidiabetic properties 2, 3. Among them, the anticancer properties of mushrooms are receiving considerable attention, and clinical trials to evaluate the effectiveness of mushrooms are increasing 4. The 5-year survival rate of non-small cell lung cancer patients treated with reishi mushrooms increased, proving the efficacy of reishi mushrooms 5. Additionally, *Agaricus bisporus* has proven efficacy in treating patients with recurrent prostate cancer by inhibiting prostate-specific antigen 6. Therefore, identification of new mushrooms with anticancer property and clinical application of their efficacy will become a new treatment method.

Mushrooms are organisms characterized by a life cycle consisting of two distinct stages: mycelium and fruiting body 7. Mycelium is a thread-like organism that grows in soil, wood, or other substrates and absorbs nutrients 7. The fruiting body is the part of the mushroom that can be found above ground and consists of a cap, stem, gills or stomata, depending on the species 7. Comparison of the composition and concentration of various compounds in fruiting bodies and mycelium revealed that mycelium has a higher content of some bioactive compounds than the fruiting body 8.

Mycelium can recently be grown in large quantities using liquid culture approaches 9. Mycelium liquid culture speeds up the colonization rate, which makes it possible to culture large quantities of mycelium at low cost 10. Mycelium liquid culture also allows for the effective production of bioactive reagents due to its ability to comply with current good manufacturing practice (cGMP) standards. Therefore, this technology is evaluated as a promising technology that can effectively produce secondary metabolites produced from mycelium.

The genus *Polyporus*, a type of wood rot mushroom, belongs to the *Polyporaceae* family 11. Currently, *Polyporous parvovarius* was identified to stimulate apoptosis and inhibit cancer cell growth 12. Despite the fact that this genus contains over 250 species, little research has been done on the anticancer abilities of other *Polyporus* species. The aim of this study is to examine whether *Polyporus ulleungus* (*P. ulleungus*) mycelium culture extract had anticancer activity. Here, we identified that extracts of *P. ulleungus* mycelium cultured in MEB medium exhibited anticancer properties and examined the underlying mechanism how it effectively kills cancer cells.

## Materials and Methods

### Procedure to prepare mycelial culture extracts

The National Institute of Biological Resources (NIBR, Incheon, Korea) provided *Polyporus ulleungus* (Accession number: NIBRFG0000513823). Table 1 lists the four media that were employed in this investigation along with their names and ingredients.

The steps for preparing mycelial culture extracts have been described in detail previously 13. Briefly, 5 mm pre-cultured mycelial disks were injected into Erlenmeyer flasks containing 700 ml of medium. To test the effect of medium alone, mycelial disks were not injected into Erlenmeyer flasks containing 700 ml of medium. Afterwards, each flask underwent a 60-day incubation period at 25°C with 150 × *g* shaking. The liquid culture medium containing mycelium and the liquid culture medium without mycelium were passed through Miracloth (475855-1R; Millipore, Burlington, MA, USA). Afterwards, the filtered culture medium was freeze-dried. The dried substance was dissolved using 100% ethanol. The dissolved solution was filtered using a 0.45m membrane filter (HAWP05000, Sigma), and the ethanol was evaporated. The leftover extract was diluted to a 500 mg/ml concentration in dimethyl sulfoxide (DMSO) (41639, Sigma).

### Cell Culture

Table 2 provides information on the eight different cell lines utilized. Cells were cultured following procedures described previously 12.

### Cell proliferation assay

Cell proliferation assay was conducted in 96-well plates with 2,000 cells per well. Each mycelium extract was diluted to a 500 μg/ml concentration. The concentration of cisplatin (PHR1624-200MG; Sigma) was diluted to a final value of 20 μM. Detailed procedures were followed as previously described 13.

### Determination of cytotoxicity of pu-MEB

Cytotoxicity assay was conducted in 96-well plates with 2,000 cells per well. pu-MEB was diluted at concentrations of 100-500 μg/ml. Detailed procedures were followed as previously described 13.

### Determination of the optimal concentration of pu-MEB

The assay to determine the optimal concentration was carried out in 96-well plates with 2,000 cells per well. Cell proliferation was assessed at 1-4 days at doses of 100-500 μg/ml. Procedures to determine the optimal concentration were followed as previously described 13.

### Apoptosis assay

As previously mentioned 12, an analysis for apoptosis was carried out using the Annexin V- FITC Apoptosis Detection Kit (556547; BD Biosciences, Franklin Lakes, NJ, USA).

### Cell cycle assay

Procedures to determine cell cycle were followed as previously described 12. Briefly, centrifugation was used to collect 1 × 10^6^ cells for 2 min at 760 × *g*. Cells were stained with 50 g/ml propidium iodide (PI, P4170-10MG; Sigma) after being fixed in 70% ethanol.

### Western blot analysis

Western blot analysis was followed as previously described 12. Briefly, utilizing a Chemidoc XRS+ system (1708265; BIO-RAD; Hercules, CA, USA), proteins were detected with SuperSignal™ West Femto chemiluminescent solution (34095; Thermo Fisher Scientific, Waltham, MA, USA). Table 3 provides information on primary and secondary antibodies used in this study.

### Statistical analyses

GraphPad Prism 7 (GraphPad Software, Boston, MA, USA) was used for the statistical analysis. To evaluate whether differences were significant, the Student's t-test or two-way ANOVA followed by Bonferroni post hoc test was used.

## Results

### Chemical screening for anticancer activity in *P. ulleungus* mycelium culture extracts

**Figure 1:**
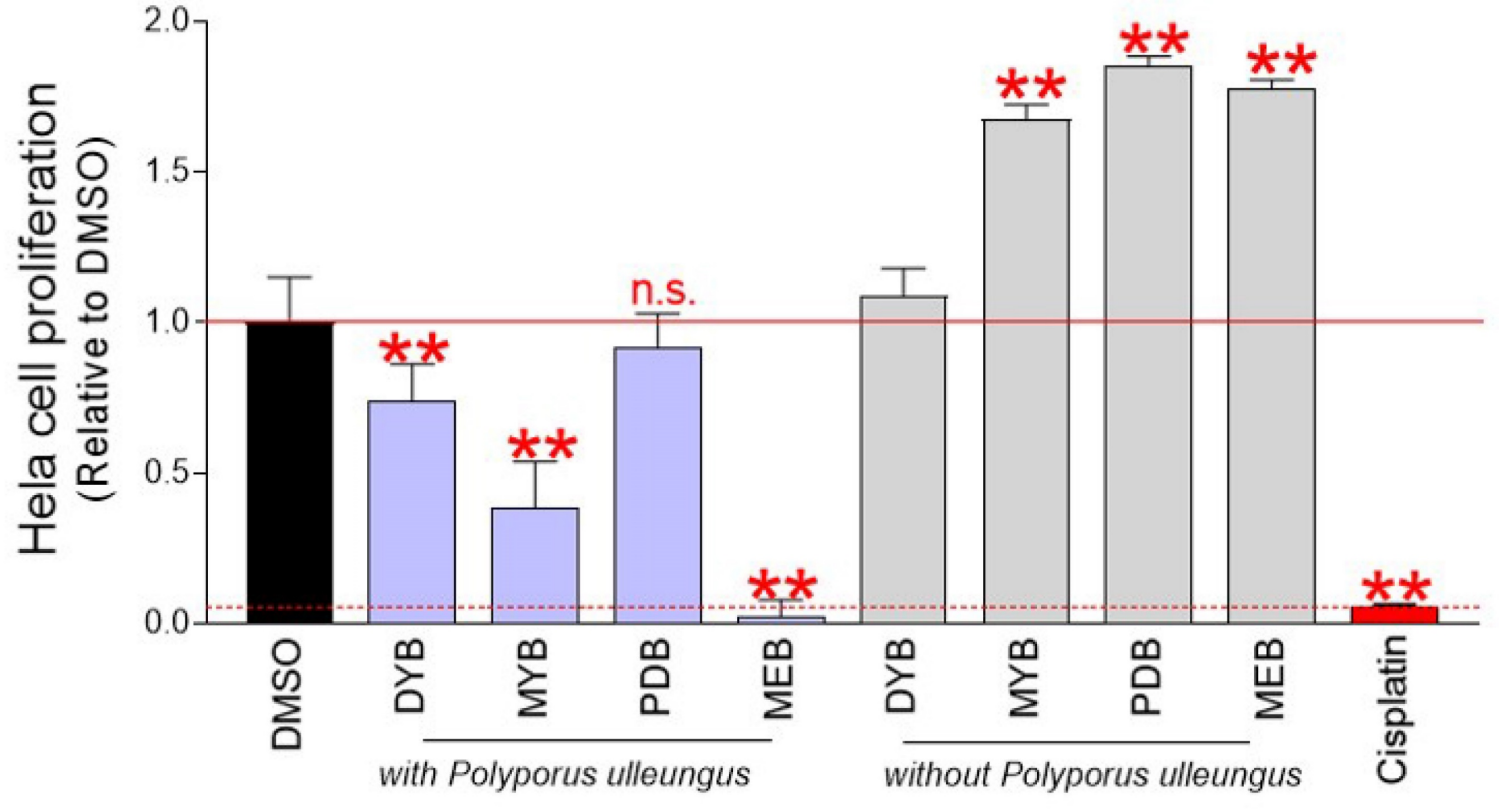
Chemical screening for anticancer activity in *P. ulleungus* mycelium culture extracts. Mycelial cultures of *P. ulleungus* grown on four different media (DYB, MYB, PDB, MEB) were extracted with four different compounds. To determine the impact of the medium on cell growth, four types of extracts were produced in medium without mycelium inoculation. Each extract was diluted to a concentration of 500 μg/ml and treated with cervical cancer-derived Hela cells. On the fourth day, the effect of each extract on cell growth was evaluated. Cisplatin, a first-line chemotherapy drug used for pancreatic, ovarian, esophageal, and cervical cancer, was used as a positive control. ** *P* < 0.01 versus control group (DMSO), *n.s*= not significant, Student's t-test. Mean ± S.D., *n* = 10.

Screening of anticancer properties of *P. ulleungus* mycelium culture extracts used a technique for counting cells based on DNA amount 14. Mycelial cultures of *P. ulleungus* grown on four different media (DYB, MYB, PDB, MEB) were extracted with four different compounds. To determine the impact of the medium on cell growth, four types of extracts were produced in medium without mycelium inoculation. Each extract was diluted to a concentration of 500 μg/ml and treated with cervical cancer-derived Hela cells. On the fourth day, the effect of each extract on cell growth was evaluated. Cisplatin, a first-line chemotherapy drug used for pancreatic, ovarian, esophageal, and cervical cancer, was used as a positive control 15. Compared with the DMSO control, mycelium culture extracts from DYB, MYB, and MEB media significantly inhibited cell proliferation (Fig. 1). However, mycelium culture extract in PDB medium did not interfere with cell growth (Fig. 1).

Identification of mycelial culture extracts with anticancer properties raised the possibility that this effect may be due to media components contained in the culture medium. To exclude this possibility, cells were treated with extracts produced in mycelium-free medium. Compared to the DMSO control, culture extracts from mycelium-free MYB, PDB, and MEB media significantly increased cell proliferation (Fig. 1). These results indicate that MYB, PDB, and MEB have nutrient-rich components that play a crucial role in increasing cell proliferation. Moreover, the culture extract of mycelium-free DYB medium was not effective in both inhibiting and inducing proliferation (Fig. 1). These results suggest that the growth inhibitory effect of mycelium extracts cultured in DYB, MYB, and MEB media is due to anticancer substances synthesized during mycelium culture rather than the media itself. Since mycelial culture extract in MEB medium (pu-MEB) inhibited cell proliferation more effectively than cisplatin (a positive control group), pu-MEB were selected for further studies (Fig. 1).

**Figure 2:**
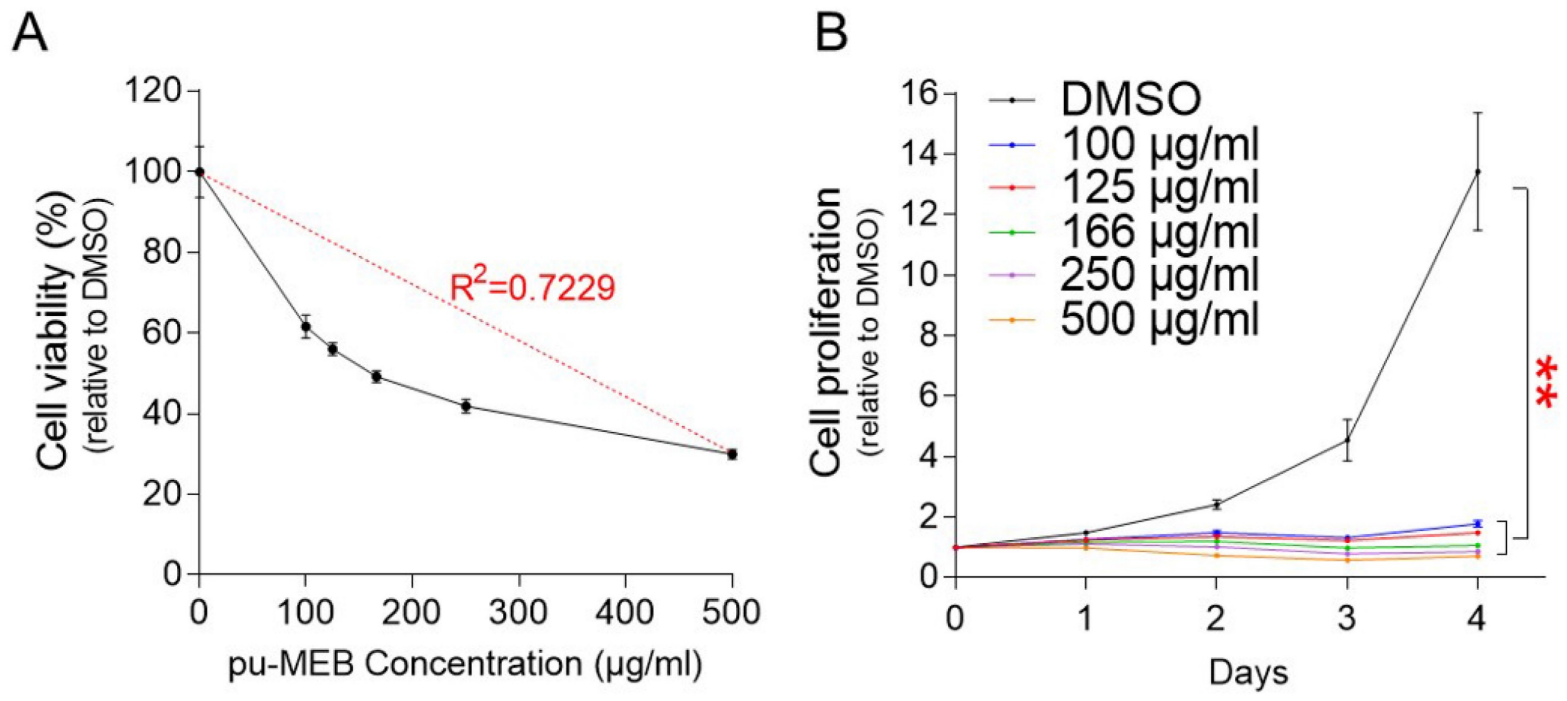
Determining the cytotoxicity and optimal concentration of pu-MEB. (A) Cell proliferation was measured at pu-MEB concentrations of 100-500 μg/ml. To assess cell viability, proliferation values at each concentration were normalized to those of DMSO. Mean ± S.D., *n* = 10. (B) Cell proliferation was measured at 1-4 days following treatment at concentration of 100 to 500 μg/ml. **P <0.01 versus control group (DMSO), two-way ANOVA followed by Bonferroni post hoc test. Mean ± S.D., *n* = 10.

The finding that the antiproliferative effect was caused by the anticancer properties of pu-MEB rather than by the effect of the medium may raise another possibility that the antiproliferative effect may be due to cytotoxicity of pu-MEB. To rule out the possibility, cytotoxicity experiment was carried out using pu-MEB at a concentration of 100-500 μg/ml. R^2^ value, coefficient of determination, was 0.7229, showing that concentration was a factor in the decreased cell viability (Fig. 2A). These results suggest that pu-MEB's ability to decrease cell growth was not a result of its cytotoxicity.

The optimal concentration of pu-MEB to effectively inhibit cell proliferation was then examined. Cell proliferation was measured at 1-4 days following treatment at concentration of 100 to 500 μg/ml. Each concentration of pu-MEB exhibited a significant decrease in cell proliferation compared to the DMSO control (Fig. 2B). As a result, the lowest concentration of pu-MEB, 100 μg/ml, was chosen for further studies.

### pu-MEB induces apoptosis and S phase arrest to reduce cancer cell growth

**Figure 3:**
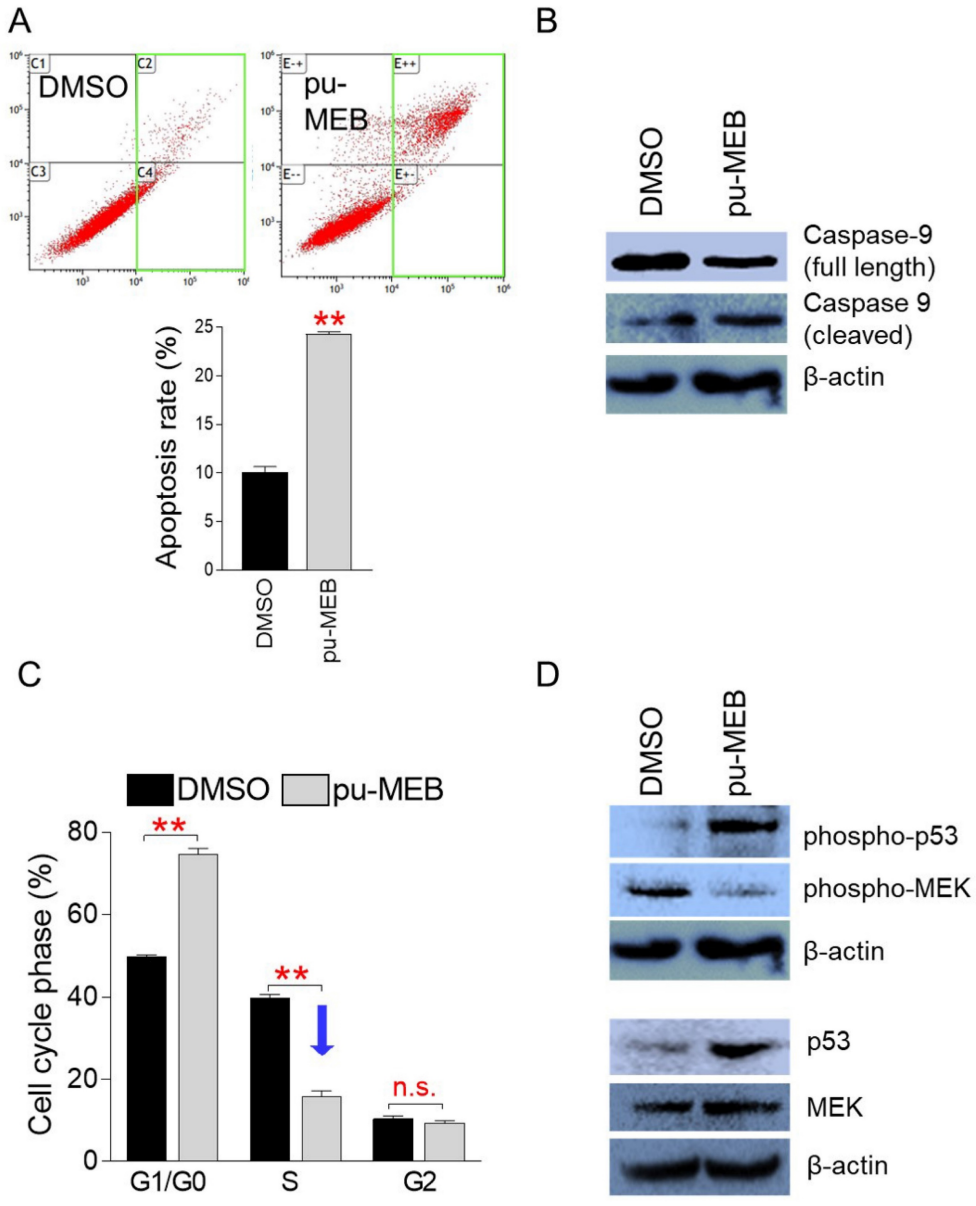
pu-MEB induces apoptosis and S phase arrest to reduce cancer cell growth. (A) To perform flow cytometric analysis of apoptotic cells, HeLa cells were treated to pu-MEB at a dose of 100 μg/ml for 2 days. Apoptotic cell populations are shown by the green square. **P < 0.01 versus control group (DMSO), student's t-test. Means ± S.D., *n* = 3. (B) Effect of pu-MEB on the expression levels of proteins that initiate the caspase cascade and induce apoptosis. Compared with the DMSO control group, full-length caspase 9 decreased and cleaved caspase 9 increased in the pu-MEB group. (C) To perform flow cytometric analysis of cell cycle, HeLa cells were treated to pu-MEB at a dose of 100 μg/ml for 2 days. **P < 0.01 versus control group (DMSO), student's t-test. Means ± S.D., *n* = 3. (D) Effect of pu-MEB on the expression levels of proteins that play important roles in the cell cycle pathway: p53, phospho-p53, MEK, phospho-MEK.

Apoptosis is a programmed cell death process known to be a crucial way to limit the uncontrolled proliferation of cancer cells 16. Thus, we investigated whether pu-MEB causes apoptosis to stop the proliferation of cancer cells. To perform flow cytometric analysis of apoptotic cells, HeLa cells were treated to pu-MEB at a dose of 100 μg/ml for 2 days. Indeed, compared to controls, pu-MEB treatment significantly increased the apoptosis rate (Fig. 3A). These findings imply that pu-MEB inhibits cancer cell proliferation by triggering programmed cell death.

Caspase 9 exists as an inactive procaspase monomer and is activated by a unique proteolytic process that causes cleavage 17. Cleaved caspase 9 processes other caspase members to initiate the caspase cascade and induce apoptosis 17. We examined the cleavage of caspase 9 to provide further evidence for the ability of pu-MEB to induce apoptosis. Compared with the DMSO control group, full-length caspase 9 decreased and cleaved caspase 9 increased in the pu-MEB group, indicating that the pu-MEB group initiated the caspase cascade leading to apoptosis (Fig. 3B).

Cancer is also characterized by unchecked cell cycle progression because of aberrant activation of cell cycle proteins 18. One of the main indicators to evaluate the anticancer properties of a candidate substance is whether it triggers cell cycle arrest, especially S phase arrest 19, 20. Therefore, we examined whether pu-MEB induces S phase arrest in cancer cells. To perform flow cytometric analysis of cell cycle, HeLa cells were treated to pu-MEB at a dose of 100 μg/ml for 2 days. Compared with cells treated with DMSO, cells treated with pu-MEB significantly upregulated the proportion of G1/G0 phase from 49.8 to 74.7%, but downregulated the proportion of S phase from 39.9 to 16.0% (Fig. 3C). According to these findings, pu-MEB caused S phase arrest in cancer cells.

Cell cycle arrest is promoted by the oncoprotein p53 (p53), allowing DNA repair 21. Additionally, the stability of p53, which is required for the initiation of cell cycle arrest, depends on phosphorylation of p53 (phospho-p53) 22. Therefore, we investigated whether pu-MEB treatment changes the expression levels of p53 and phospho-p53. pu-MEB significantly increased the expression of p53 and phospho-p53, demonstrating the underlying mechanism of pu-MEB-mediated S phase arrest (Fig. 3D).

Mitogen-activated protein kinase kinase (MEK) acts as a key component of the MAP kinase signal transduction pathway. Phospho-MEK is an active form of MEK and is a key regulator of S phase entry signals 23, 24. Therefore, we examined the expression level of MEK and phospho-MEK. pu-MEB did not markedly reduce the expression of MEK, but markedly reduced the expression of phopsho-MEK (Fig. 3D). These results indicate pu-MEB-mediated S phase arrest through reduced expression of phopsho-MEK.

### pu-MEB inhibits the proliferation of lung cancer-derived cancer cells

**Figure 4:**
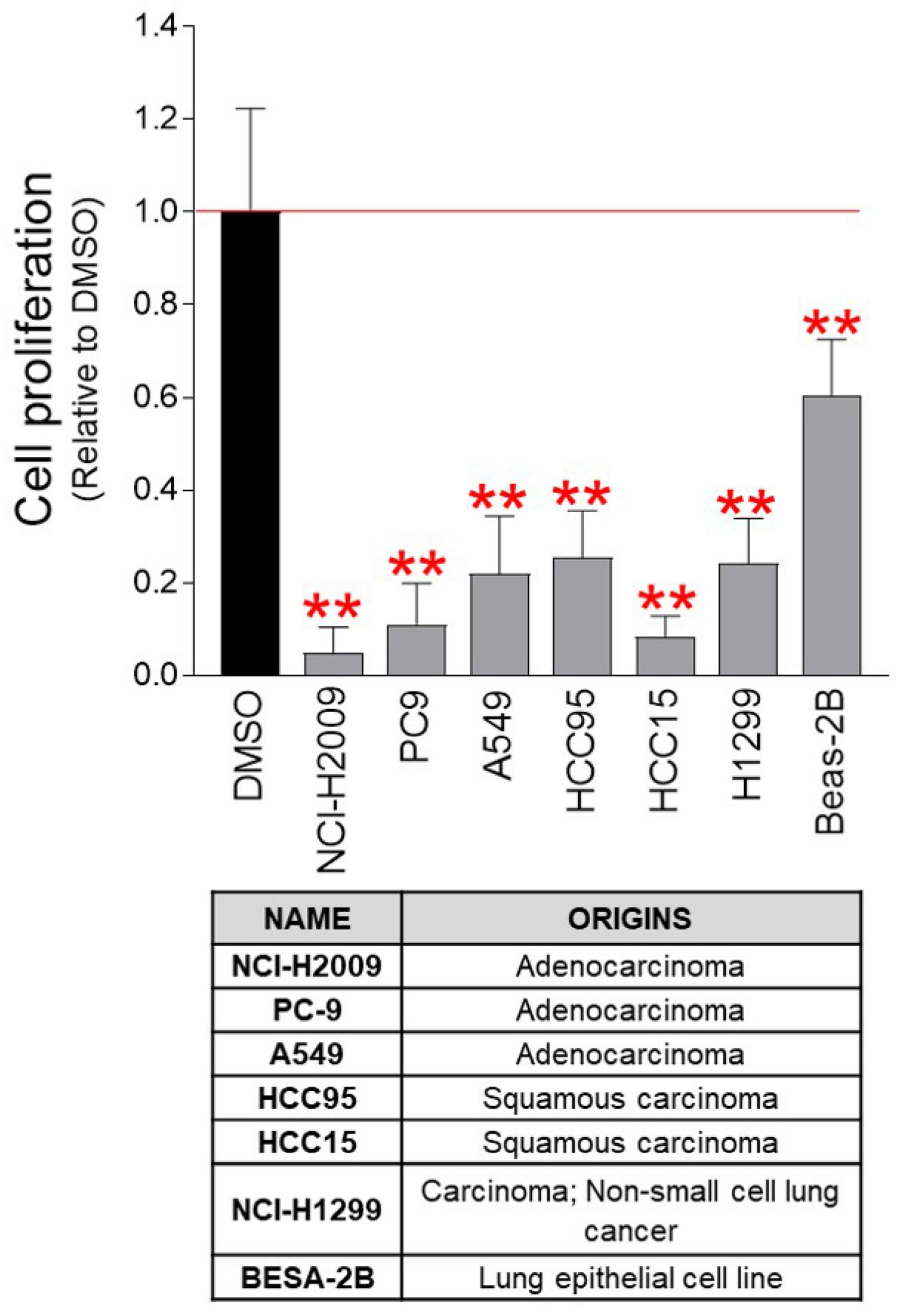
pu-MEB inhibits the proliferation of lung cancer-derived cancer cells. Seven lung cancer-derived cells were treated with pu-MEB at a concentration of 100 μg/ml. On the fourth day, the inhibitory effect on cell proliferation was assessed. **P < 0.01 versus control group (DMSO), student t-test. Means ± S.D., *n* = 10.

As the anticancer effect of pu-MEB was confirmed in cervical cancer-derived Hela cells, we examined whether the pu-MEB had anticancer properties against various cancer cell lines. Seven lung cancer-derived cancer cell lines (NCI-H2009, PC-9, A549, HCC95, HCC15, NCI-H1299, and BESA-2B) were used to determine the effectiveness of pu-MEB. Seven lung cancer-derived cancer cell lines treated with pu-MEB exhibited significantly less proliferation than cells treated with DMSO (Fig. 4). These findings imply that the anticancer characteristics of pu-MEB are effective not only for cervical cancer-derived Hela cells, but also for lung cancer-derived cancer cells.

## Discussion

Mushrooms produce physiologically active secondary metabolites such as polysaccharides, lectins, terpenoids, and alkaloids in response to various external stimuli 25. Secondary metabolites have low toxicity and no side effects, so they are used for anticancer, antibacterial, and antidiabetic purposes. Numerous clinical trials are being conducted to assess the efficiency and safety of secondary metabolites 4. In this study, *P. ulleungus* mycelium was cultured in four media (DYB, MYB, PDB, MEB) and the anticancer ability of mycelium culture extract was investigated. Significant anticancer activity was demonstrated by mycelia grown on DYB, MYB and MEB media. However, extracts grown in PDB did not show anticancer property. Differences in media composition can explain the underlying mechanism for these results. DYB, MYB and MEB media contain yeast extract, but PDB medium does not contain yeast extract. Thus, we propose that yeast extract is essential for *P. ulleungus* mycelium to produce secondary metabolites that exhibit anticancer activity. Furthermore, among the mycelium culture extracts grown in DYB, MYB, and MEB media, the mycelium culture extract grown in MEB medium showed the most potent anticancer activity. To find the cause of these results, the components of DYB, MYB, and MEB media were analyzed. DYB medium contains yeast extract and dextrose. DYB medium contains two components: yeast extract and glucose. MYB medium contains two components: yeast extract and malt extract. However, MEB medium contains four components: yeast extract, malt extract, dextrose, maltose. Moreover, maltose is an ingredient that is not included in DYB and MYB, but is only included in MEB medium. Therefore, we propose that maltose is essential for *P. ulleungus* mycelia to synthesize secondary metabolites that exhibit anticancer property. Taken together, this is the first study to demonstrate medium conditions for *P. ulleungus* mycelium to synthesize metabolites with anticancer property. If the components that make up MEB medium can be improved using methods such as waste medium analysis, *P. ulleungus* mycelium will be able to synthesize metabolites with more powerful anticancer properties.

Eukaryotic cells have the ability to eliminate their own cells through apoptosis 26. Because cancer cells have the characteristic of avoiding apoptosis, apoptosis has been used as a successful strategy to eliminate cancer cells 16. Here, we identified that pu-MEB initiated the caspase cascade by decreasing the inactive caspase 9 form and increasing the active caspase 9 form, ultimately inducing apoptosis. Dysregulation of cell cycle progression is another characteristic of cancer cells 18. Because cancer cells have the characteristic of uncontrolled cell cycle progression, regulating cell cycle progression have been targeted as strategies to treat the uncontrolled proliferation of malignant tumor cells 18. Here, we found that pu-MEB regulates the cell cycle progression of cancer cells. pu-MEB activated p53, which controls checkpoints throughout the cell cycle from G1 phase to cytokinesis. In addition, pu-MEB inhibited the activity of MEK, which is activated in S phase and promotes DNA synthesis, thereby inducing S phase arrest. Because apoptosis induction and S phase arrest act as a double-edged sword in effectively eliminating cancer cells, we propose that the use of pu-MEB as a chemotherapy agent should be considered a priority.

Mycelium liquid culture allows mycelium to be grown in liquid without using conventional agar plates 27, 28. Because mycelium quickly colonizes liquid media, the scale of mycelium liquid culture can be expanded up to 10,000 liters. The expanded culture scale enables the production of large quantities of secondary metabolites at a lower cost 9. Here, we identified that *P. ulleungus* mycelium cultured in MEB generated metabolites with anticancer properties. However, because liquid culture was carried out in a laboratory environment, there is a limit to the amount of metabolites produced at one time. If the large-scale mycelium liquid culture method can be improved by altering process variables including dissolved oxygen, temperature, stirring speed, and pH, the amount of metabolites generated per one liquid culture could reach commercial production levels.

Paclitaxel, a well-known anticancer drug, was extracted from the bark of *Taxus brevifolia* through fractionation and purification 29. Penicillin, a well-known antibiotic, was also extracted from the mold *Penicillium chrysogenum* through fractionation and purification 30. Previous studies found that *P. parvovarius* mycelium culture extract exhibits anticancer efficacy 12. Then, active ingredients were obtained through a fractionation/purification process and identified as 3,4-dihydroxybenzaldehyde (Protocatechualdehyde, PCA). PCA showed anticancer activity similar to* P. parvovarius* mycelium culture extract, confirming PCA as the active ingredient 12. In this study, we identified that pu-MEB exhibits anticancer efficacy, but did not confirm which active component of pu-MEB exhibits anticancer efficacy. Therefore, future research will need to find active ingredients that exhibit anticancer activity in pu-MEB through separation, purification, and structural analysis. If an active ingredient with anticancer efficacy is discovered through this process, the ingredient can be used as a new anticancer agent to treat cancer.

In summary, we identified that *P. ulleungus* mycelia cultured in MEB medium (pu-MEB) significantly reduced cancer cell growth by triggering apoptosis and S phase arrest. The efficacy of pu-MEB was not limited to cervical cancer-derived Hela cells, but was also effective against lung cancer-derived cancer cells. This potent anti-cancer candidate can be generated in large quantities at low cost and can be used clinically to treat cancer.

## Figures and Tables

**Table 1:** Four media that were employed in this investigation.

Media name	Ingredients	Company name	Catalogue number	Concentration
DYB	• yeast extract	Becton Dickinson, Franklin, NJ, USA	212750	2g/l
	• dextrose	Becton Dickinson	215530	20 g/l
MYB	• yeast extract	Becton Dickinson	212750	2g/l
	• malt extract	Becton Dickinson	218630	20 g/l
PDB	• dextrose	Becton Dickinson	215530	20 g/l
	• potato starch	Sigma, Saint Louis, MO, USA	S2004	4 g/l
MEB	• yeast extract	Becton Dickinson	212750	2g/l
	• malt extract	Becton Dickinson	218630	6 g/l
	• dextrose	Becton Dickinson	215530	6 g/l
	• maltose	Becton Dickinson	216830	1.8 g/l

**Table 2:** Information on the seven different cell lines utilized.

Cell line name	Company name	Catalogue number
HeLa cells	Sigma	93021013
NCI-H2009	American Type Culture Collection (ATCC), Manassas, VA, USA	CRL-5911™
PC-9	Sigma	90071810
A549	ATCC	CRL-185™
HCC95	Sigma	CLL1220
HCC15	Deutsche Sammlung von Mikroorganismen und Zellkulturen, Braunschweig, German	ACC 496
NCI-H1299	ATCC	CRL-5803™
BEAS-2B	ATCC	CRL-9609™

**Table 3:** Information on primary and secondary antibodies used in this study.

Antibody name	Company name	Catalogue number	Dilution in PBS
caspase 9 antibody	Cell signaling technology, Danvers, MA, USA	9502s	1:500
phospho-p53 antibody	Santa Cruz Biotechnology, Dallas, TX, USA	sc-377561	1:500
phospho-MEK antibody	Cell signaling technology	9121s	1:500
p53 antibody	Abclonal, Woburn, MA, USA	A19585	1:500
MEK antibody	Cell signaling technology	9122s	1:500
HRP-conjugated β-actin	Santa Cruz Biotechnology	sc-47778	1:1,000
anti-mouse HRP-conjugated antibody	Santa Cruz Biotechnology	sc-516102	1:1,000
anti-rabbit HRP-conjugated antibody	Santa Cruz Biotechnology	sc-2357	1:1,000
